# Current Trends in Preservation Rhinoplasty

**DOI:** 10.1093/asjof/ojaa003

**Published:** 2020-02-01

**Authors:** Rollin K Daniel, Aaron M Kosins

## Abstract

Recently there has been a dramatic acceptance of the preservation principle in rhinoplasty surgery. Surgeons worldwide now preform preservation rhinoplasty, which has led to an expanding list of indications and techniques. Most rhinoplasty surgeons have accepted the fundamental principle that preservation is better than resection and that a natural result is superior to a fabricated or reconstructed structure, especially with regards to the nasal dorsum. Currently, the main emphasis is on defining the indications/contraindications, technical refinements, and minimizing complications. This paper provides an overview of the current trends in preservation rhinoplasty. In the 2 years following publication of the Editorial, “The Preservation Rhinoplasty: A New Rhinoplasty Revolution,” ^1^ there has been a dramatic acceptance of the preservation principle. Numerous surgeons throughout the world are preforming preservation rhinoplasty, which has led to an expanding list of indications and techniques. The majority of rhinoplasty surgeons have accepted the fundamental principle that preservation is better than resection, and that a natural result is superior to a fabricated or reconstructed structure, especially as regards the nasal dorsum. Currently, the main emphasis is on defining the indications/contraindications, technical refinements, and minimizing complications. The present paper is an overview of the current trends in preservation rhinoplasty.

## COMPOSITION

Preservation rhinoplasty (PR) is composed of the following 3 parts: (1) elevating the soft tissue envelope (STE) in a subperichondrial–subperiosteal plane while preserving the scroll ligament complex; (2) preserving the nasal dorsum without creating an open roof deformity; and (3) maintaining the alar cartilages and achieving the desired shape using sutures instead of excision. It should be noted that PR refers to these 3 components, one of which is dorsal preservation—the 2 terms should not be used interchangeably.

## SOFT TISSUE ENVELOPE

Currently, most surgeons elevate the soft tissue envelope in the sub-SMAS plane, as it is relatively avascular and less disruptive than the previously utilized subcutaneous plane. However, the sub-SMAS dissection is still associated with significant postoperative swelling, numbness, prolonged scar remodeling, and induration. Long-term thinning of the STE is a major concern as noted by Tardy and recently demonstrated by Toriumi.^2^ In contrast, elevating the STE with a continuous subperiochondrial–subperiosteal dissection (herein after SSD) results in minimal swelling, near normal sensation, minimal scar remodeling, and avoidance of long-term thinning of the STE.^3^ Elevation of the STE as a single sheet is critical to minimizing both short and long-term problems. In addition, it is possible to preserve and/or to restore the nasal ligaments including Pitanguy’s, the scroll ligament complex, and the intercrural ligament. When joining the subperichondrial dissection pockets between the alar and upper lateral cartilages, it is possible to keep the scroll ligament complex intact. Subsequently, it’s reattachment to the underlying soft tissue at the end of the surgery has 3 distinct benefits: (1) creation of a distinct surface aesthetic line; (2) closure of dead space; and (3) stabilization of the internal valve. Preservation or repair of Pitanguy’s ligament improves tip projection, shortens the infralobular curve, and accentuates supratip break.^4^

As previously reported,^5^ ultrasound assessment using a 25-MHz transducer allows the surgeon to assess the composition of the STE preoperatively and postoperatively over a sequential period.^6^ Essentially, one can measure the thickness of the dermis and the underlying soft tissue. In patients with a normal healing progression, 2 distinct layers representing the dermis and the underlying subcutaneous tissues are visualized. In patients with scar formation, one can often see abnormal thickness of the STE as early as 6 weeks, which on sonogram reveals a third, disorganized scar tissue layer that can be treated with direct injections of 0.1ml of Kenalog 10 under ultrasonic guidance. Of particular interest is the different effect of “plane of dissection” on healing of the STE. Following a sub-SMAS dissection, the dermis and the soft tissue thickness becomes swollen and thickens on sonogram. Beginning at the first month one sees peak swelling followed by gradual reorganization and eventual thinning of the soft tissue. Sonograms at the keystone area demonstrate that swelling in both layers continues to decrease until 9 months postoperatively. In contrast, sonograms done at similar time periods in patients having a subperichondrial elevation of the STE demonstrate that swelling in both layers reaches a steady state at approximately 3 months in the keystone area with less thinning of the soft tissues. Since each patient serves as their own control, the changes in STE architecture are readily apparent. Studies in this area of research are currently ongoing by the senior author.

One of the surprising benefits of dorsal preservation is the ability to limit the extent of dorsal skin dissection as there is no need to do a wide exposure to enable excision and reconstruction. Gola in his report of a 1000 cases of DP was a strong advocate of no dorsal skin undermining as it insured protection of both the skin and the nasal musculature.^7^ Recently, Goksel and others have adopted a more limited skin dissection in many of their DP cases.^8^ In fact, Finocchi does no dorsal skin undermining in approximately 30% of cases.^9^ Obviously, the ability to avoid skin undermining reduces the risk of visualizing dorsal deformities while eliminating the need for fascia grafts and the risk of long-term thinning. The senior author (A.M.K.) performs no skin undermining of the dorsum when patients have an overprojected nose with minimal or no hump. A high subdorsal septal strip is removed and osteotomies are done with osteotomes via an external approach to release the osseocartilaginous vault from the midface.

## DORSAL PRESERVATION

**Figure 1.**
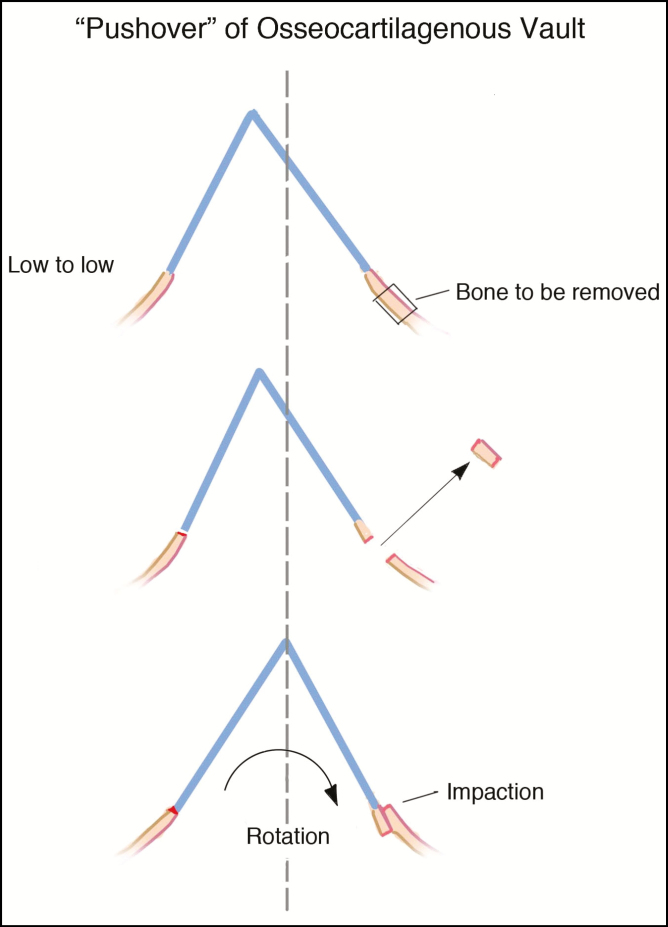
This diagram demonstrates a “push over” of the osseocartilaginous vault. After removal of a septal strip, low to low and transverse osteotomies are performed bilaterally as well as a radix osteotomy. On the deviated (long) side, a strip of lateral nasal bone is removed at the face of the maxilla. On the short side, a low osteotomy is performed without bone removal. Once the osseocartilaginous vault is mobilized the nose is pushed over and impacted on the long side.

To understand the current trends of dorsal preservation (DP), it is important to understand its evolution. The concept of preserving the dorsum while reducing the height of the dorsal bridge is not new. Goodale reported his experience with an aesthetic case of dorsal reduction that preserved the dorsum using a combination of a high septal strip excision and push down technique and 2 years later, he followed up this case report with a series of 22 post-traumatic rhinoplasties using a similar “push over” technique.^10^ Although used sporadically over the next half century, the concept of the push down operation was resurrected by Cottle in 1954. His technique differed from Goodale as he favored a tripartite septal excision consisting of the following: (1) a 4 mm wide vertical strip at the quadrangular cartilage/ethmoid plate junction; (2) an 8–12 mm triangular excision of perpendicular plate of ethmoid (hereinafter PPE); and (3) a longitudinal strip excision from the inferior border of the mobilized quadrangular cartilage.^11^ For several reasons, including the rise of the open approach, the Cottle procedure was not accepted by the majority of rhinoplasty surgeons, although it continued to be done in multiple centers throughout the world. Most surgeons followed the Joseph method of resecting the dorsal hump, which created an “open roof” deformity. Currently, surgeons do some form of mid-vault reconstruction with either spreader grafts or spreader flaps. Despite the popularity of dorsal resection, a small number of surgeons continued to do DP. The most notable proponents were Gola and Saban who favored a high subdorsal septal strip excision, which allowed the dorsal convexity to flatten.^12^ Mobilization and lowering of the bony nasal pyramid was achieved with either a push down or let down procedure. A push down procedure lowers the bony pyramid into the pyriform aperture. A let down procedure involves removal of a strip of bone laterally at the junction of the nose with the maxilla. In a let down, the bony pyramid sits on the ascending process of the maxilla. These two methods of lateral osteotomies are often combined in the treatment of the asymmetric, crooked nose where a let down strip excision is done on the longer angulated side and a push down on the shorter vertical side (Figure 1).

**Figure 2.**
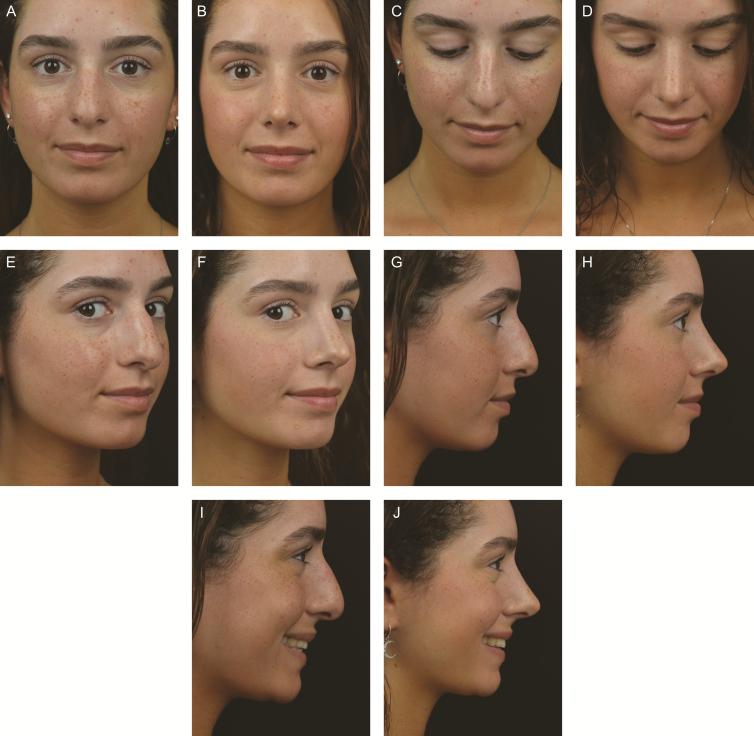
A 22-year-old female who underwent a cartilage pushdown after bony cap modification. (A, C, E, G, I) Preoperative and (B, D, F, H, J) 1-year postoperative photographs are shown. The patient has an ideal cartilaginous vault with wide nasal bones and a hump of approximately 3.5 mm. After the removal of the bony cap with piezoelectric surgery, a 3-mm strip of subdorsal septum is removed. The cartilage vault is conservatively disarticulated at the lateral keystone area from the nasal bones. Once mobilization has occurred, 5-0 PDS sutures are used at multiple points to sew the preserved cartilaginous vault down to the underlying septum. Piezoelectric medial oblique, transverse and low-to-low osteotomies are performed to narrow the bony dorsum and bone base. In this way, the cartilage vault is preserved and the bones are narrowed. The dorsal aesthetic lines have been improved and the bony vault narrowed. The profile line is improved without opening the middle vault.

Thus, what are the current trends in DP? First, one can convert osseocartilaginous humps into pure cartilaginous humps by removing the bony cap. Removing or reshaping the bony vault creates a more pliable cartilage vault that is more favorable for dorsal preservation. It should be noted that both Cottle and Saban removed small bony humps prior to doing a push down procedure. In 1975, Barelli emphasized that combining the removal of small amount of the bony hump with a push down procedure “does not violate the principles of a push-down procedure provided an open roof is not created as evidenced by removal of mucous membrane.” ^13^ Recently, Ferreira et al have revived this procedure as the “Spare Roof Technique” (SRT), using either a closed or open approach.^14^ It’s essential 4 steps are as follows: (1) a longitudinal cut through the dorsal septum from ASA to PPE thus releasing the cartilaginous dorsum from the septum; (2) decreasing the dorsal hump by serial 1 mm septal resections; (3) ostectomy of the caudal end of the bony hump; and (4) suture fixation of the cartilaginous hump to the septum. They validated the aesthetic and functional benefits of the technique in a follow-up study.^15^ They felt that the primary indication was straight Caucasian noses with a hump smaller than 5 mm. It should be noted that resection of a portion of the PPE is not an integral part of the dorsal reduction as it is in both the Cottle and Saban procedures. In the senior author’s experience (A.M.K.), *cartilage conversion techniques* have been extremely valuable in dealing with nasal humps under 3 mm and certain asymmetries. The modifications which have been extremely valuable include the following: (1) an open approach for assessment and exposure; (2) the use of Piezo electric rasps; (3) removal and/or modification of the bony cap before septal strip excision; (4) precise osteotomies if needed after pushdown of the cartilaginous vault; and (5) precise suturing of the osseocartilaginous vault down to dorsal septum thereby controlling width and symmetry. This procedure is intuitively simpler and structurally very stable because the nose has not been separated from the face. Figure 2 is a case study demonstrating dorsal preservation after bony modification and cartilage conversion.

**Figure 3.**
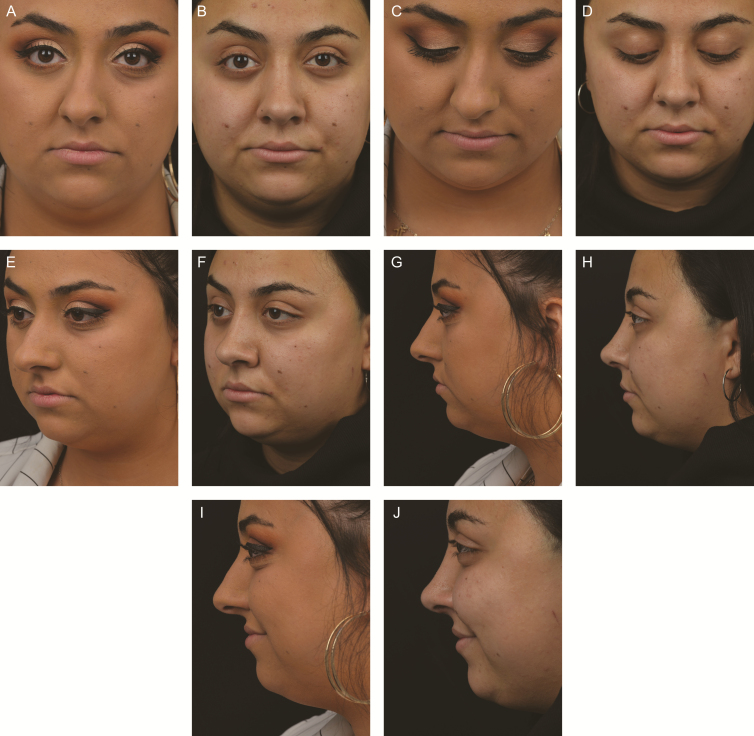
A 23-year-old female with asymmetric, developmental deviation of the nose. (A, C, E, G, I) Preoperative and (B, D, F, H, J) 9-month postoperative photographs are shown. This patient has an ideal osseocartilaginous vault with a small hump and straight line deviation to the right. The quadrangular cartilage was disarticulated from the anterior nasal spine, vomer and perpendicular plate of ethmoid creating a “swinging door.” A “push over” of the osseocartilaginous vault was performed and profile line lowered by removing a 4-mm low septal strip and performing low to low and transverse osteotomies bilaterally as well as a radix osteotomy. On the long (left) side, a 3-mm strip of lateral nasal bone was removed at the face of the maxilla. On the short side, a low osteotomy was performed without bone removal. Once the osseocartilaginous vault was mobilized the nose was pushed over and impacted on the long side. The caudal septum was then reattached to the anterior nasal spine via a drill hole. The dorsal aesthetic lines remain idea and the osseocartilaginous vault has been centralized on the face. In effect, the nose was straightened without opening the middle vault or performing multiple open and closed book ostetomies. Interestingly, the nasal tip deviation is fixed once the septum is placed in the midline.

Second, is adoption of a modified Cottle procedure, especially in cases of high septal deviation or deviated noses. Multiple surgeons continue to do a standard Cottle procedure with its tripartite septal resection.^16^ The primary problem that they encounter is the appearance of a residual hump. Other surgeons, including Saban and the senior author (A.M.K.) do both techniques of longitudinal strip excisions or a Cottle depending on relative indications. Saban reserves the Cottle procedure for difficult post-traumatic cases with major septal deformities requiring septal cartilaginous resections that preclude a subdorsal strip excision. He considers the negatives of the Cottle procedure to be a longer operative time and greater difficulty in dorsal positioning. Recently, Finocchi has introduced a modified Cottle procedure, which he calls the “Simplified Preservation Quick Rhinoplasty” (SPQR thus reflecting his Roman location).^17^ It should be noted that he had done 150 DP operations using the Saban technique before switching to his Cottle modification. The change was prompted by the need for dealing with high septal deviations particularly in the bony portion. Finocchi’s modifications from the standard Cottle are as follows: (1) the vertical cut in the septum is an incision without the requisite excision of a vertical strip; (2) the location of the vertical strip is not mandated by the quadrangular-PPE junction, but rather by the desired dorsal pivot point in the keystone area; and (3) there is no need for paraseptal medial osteotomies separating the nasal bones from the septum and each other. In over 100 consecutive cases, Finocchi has found the procedure to be extremely flexible and partiualry valuable in dealing with deviated noses. The senior author (A.M.K.) has done approximately 50 Modified Cottle procedures over the past year and considers the current indications to be as follows: (1) asymmetric developmental deviation of the nose with an ideal dorsum that is simply shifted off the midline; and (2) high septal deviations. This technique is very versatile as 3 vectors are controlled including impaction of the osseocartilaginous vault, rotation of the bony dorsum, and a swinging door septoplasty with cartilaginous repositioning. This is in contrast to the high septal strip method with 2 vectors of movement where the osseocartiliginous vault is impacted and any rotation is of the complete vault without separation of the bony and cartilaginous components. Thus, each procedure has distinct advantages and is selected based on the patient’s deformity. Figure 3 is a case study for a modified Cottle procedure.

## ALAR CARTILAGE PRESERVATION

Traditionally, surgeons achieved the desired tip shape using a combination of excision, incision, sutures, and grafts. Although results were good initially, a significant percentage of these cases deteriorated over time. However, the adoption of tip suturing and structural support using various columellar struts, septal extension grafts, and tongue in groove procedures resulted in dramatically improved intermediate term results with maintenance of projection and fewer tip deformities. However, PR advances tip surgery even further by preserving virtually the entire alar cartilage, which enhances function and reduces potential problems. The combination of a subperichondrial exposure, preservation of ligaments, and maintenance of a completely intact alar cartilage represents a dramatic new advance in tip surgery.

**Figure 4.**
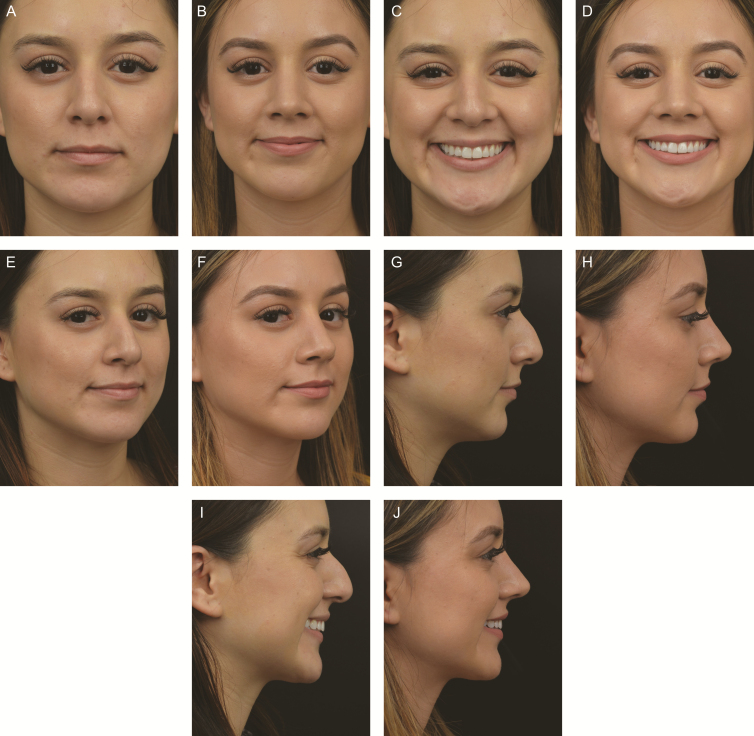
A 23-year-old female who underwent a total preservation rhinoplasty. A subperichondrial-subperiosteal dissection was performed of the nose with preservation of Pitanguy and scroll ligaments. (A, C, E, G, I) Preoperative and (B, D, F, H, J) 15-month postoperative photographs are shown. No alar cartilage was removed and a high septal strip pushdown was performed. Because the patient had very weak cartilage, a septal extension graft was used to project the lower lateral cartilages against the soft tissue envelope to gain projection and definition. Cranial tip sutures were used with a 2.5-mm lateral steal. The combination of tip sutures, a lateral steal procedure, and septal extension graft effectively tensioned the tip. Tip definition with adequate projection and rotation was gained without removal or transection of alar cartilage. All ligaments were preserved. In effect, nothing was removed from the osseocartilaginous vault or alars.

Currently, there are two techniques for alar preservation (hereinafter AP)—complete and “incise and slide.” The complete technique has its origin in the pioneering work of Arturo Regalado-Briz.^18^ His technique consists of a tip suturing technique with a columellar strut resulting in a symmetrical unified tip complex all done *without any cephalic lateral crus excision.* He argued strongly against alar excision as it leads to scar formation, structural distortion, and functional sequelae. It should be noted that he did make small peri-domal cartilage excisions similar to Davis’ “fingernail clipping” excisions. Subsequently, the senior author (A.M.K.) has incorporated Davis’ septal strut and lateral tensioning technique to achieve tip shaping with no alar cartilage excision or incisions—truly *complete alar preservation*. The technique consists of the following important steps: (1) attachment of a septal extension graft to the caudal septum either end to end or side to side; (2) domal creation sutures to create tip definition; and (3) lateral crural steal that shortens and tensions the lateral crus. These 3 steps create a rigid tip complex tensioned at all 3 legs of the tripod. Note: there is no excision of alar cartilages, either cephalically nor paradomal, nor are there any transections as frequently done in the middle crus. When it is not possible to do a complete alar preservation, one can perform an *incise and slide* procedure based on Ozman’s technique.^19^ The procedure consists of the following steps: (1) cephalic trim leaving at least an 8 mm rim strip; (2) undermining of the lateral crura; (3) suturing of the cephalic island under the remaining lateral crura without disrupting the longitudinal scroll ligament; and (4) reattachment of the vertical scroll ligament. Figure 4 is a case study of a patient with total preservation of the soft tissue envelope, dorsum and alar cartilages.

## CONCLUSIONS

Preservation rhinoplasty is a paradigm shift in rhinoplasty philosophy. While the techniques continue to improve, the philosophy remains the same – to preserve and to reshape the existing nasal structures. Not all patients benefit from PR, and some patients only benefit from partial PR, but preserving anatomy allows for a more intuitive operation and natural results.

## References

[1] Daniel RK The preservation rhinoplasty: a new rhinoplasty revolution. Aesthet Surg J. 2018;38(2):228-229. 29319790 10.1093/asj/sjx258

[2] Toriumi D *Structure Rhinoplasty: Lessons Learned in 30 Years*. Chicago: DMT Solutions; 2019:1645-1683.

[3] Kosins AM. Subperiochondrial dissection and the Scroll Ligament Complex (SLC), 2019, Rome, Italy.

[4] Cakir B. Aesthetic Septorhinoplasty. Istanbul, Turkey: Springer; 2015.

[5] Kosins AM, Obagi ZE Managing the difficult soft tissue envelope in facial and rhinoplasty surgery. Aesthet Surg J. 2017;37(2):143-157. 27965218 10.1093/asj/sjw160

[6] Kosins AM Comprehensive diagnosis and management of the difficult rhinoplasty patient: applications in ultrasonography and treatment of the soft-tissue envelope. Facial Plast Surg. 2017;33(5):509-18. 28962057 10.1055/s-0037-1606639

[7] Gola R, Nerini A, Laurent-Fyon C, Waller PY Conservative rhinoplasty of the nasal canopy. Ann Chir Plast Esthet. 1989;34(6):465-75. 2482688

[8] Goksel A. Open approaches in preservation Rhinoplasty. Preservation Rhinoplasty Meeting, 2019, Rome, Italy.

[9] Finnochi V. SPQR technique. Preservation Rhinoplasty Meeting, 2019, Nice, France.

[10] Goodale JL The correction of old lateral displacements of the nasal bones. Boston Med Surg J. 1901;20:538-9.

[11] Cottle MH Nasal roof repair and hump removal. AMA Arch Otolaryngol. 1954;60(4):408-14. 13196786 10.1001/archotol.1954.00720010420002

[12] Saban Y, Daniel RK, Polselli R, Trapasso M, Palhazi P Dorsal preservation: the push down technique reassessed. Aesthet Surg J. 2018;38(2):117-131. 29319787 10.1093/asj/sjx180

[13] Barelli PA Long term evaluation of “push down” procedures. Rhinology. 1975;13(1):25-32. 1224123

[14] Ferreira MG, Monteiro D, Reis C, et al. Spare roof technique: a middle third new technique. Facial Plast Surg. 2016;32(1):111-6. 26862972 10.1055/s-0035-1570503

[15] Santos M, Rego ÂR, Coutinho M, Sousa CAE, Ferreira MG Spare roof technique in reduction rhinoplasty: prospective study of the first one hundred patients. Laryngoscope. 2019;129(12):2702-2706. 30628092 10.1002/lary.27804

[16] Montes BJJ, Lopez UF, Del Moral VMB, et al. Influence of the lowering of the nasal pyramid technique (let down) on the middle third face. Otorrinolaringologia. 2015;60(4):234-42.

[17] FInnochi V. SPQR technique. Preservation Rhinoplasty Meeting. Rome, Italy; 2019.

[18] Regalado-Briz A, Byrd SH Aesthetic rhinoplasty with maximum preservation of alar cartilages: experience with 52 consecutive cases. Plast Reconstr Surg. 1999;103(2):671-80; discussion 681-2. 9950559 10.1097/00006534-199902000-00048

[19] Ozmen S, Eryilmaz T, Sencan A, et al. Sliding alar cartilage flap: a new technique for nasal tip surgery. Ann Plast Surg. 2009;63(5):480-5. 19801923 10.1097/SAP.0b013e31819538a8

